# Synthesis of quaternized magnetic chitosan and adsorption performance for methyl orange from aqueous solution†

**DOI:** 10.1039/d5ra02862k

**Published:** 2025-06-23

**Authors:** Kai Wang, Zewen Song, Ziyi Xu, Yang Xi, Yuwei Cui, Haijun Zhou

**Affiliations:** a School of Materials Science and Engineering, Jiangsu University of Science and Technology Zhenjiang 212100 China zhouhaijun@just.edu.cn

## Abstract

Chitosan is considered an excellent carrier material with great potential due to its good biocompatibility, abundant reserves, and high chemical reactivity. However, chitosan's chemical instability and low mechanical strength limit its applications. In this study, quaternized magnetic chitosan (QMCS) was prepared by modifying magnetic chitosan microspheres (MCS) with quaternary ammonium. The obtained adsorbent was characterized using scanning electron microscopy (SEM), Fourier transform infrared (FTIR) spectroscopy, X-ray diffraction (XRD), and X-ray photoelectron spectroscopy (XPS). The effect of pH value and adsorbent dosage on the adsorbent's performance was investigated. The experimental results indicated that QMCS exhibited superior adsorption performance for methyl orange (MO) compared to MCS. At 298 K and pH 4, the adsorption capacity of QMCS for a 125 mg L^−1^ methyl orange (MO) solution reached 486.13 mg g^−1^, with a removal efficiency of 99.38%. The adsorption behavior of the adsorbent towards MO was in good agreement with the Langmuir isothermal model and the quasi-second-order kinetics model. The mechanism of adsorption may be attributed to electrostatic interactions and ion exchange. The synthesis of QMCS was simple, environmentally friendly, and has significant potential for water pollution treatment.

## Abstract 2

In this study, QMCS was prepared by modifying magnetic chitosan microspheres with quaternary ammonium, and its adsorption capacity for a 125 mg L^−1^ methyl orange (MO) solution reached 486.13 mg g^−1^ at 298 K and pH 4.

## Graphical abstract

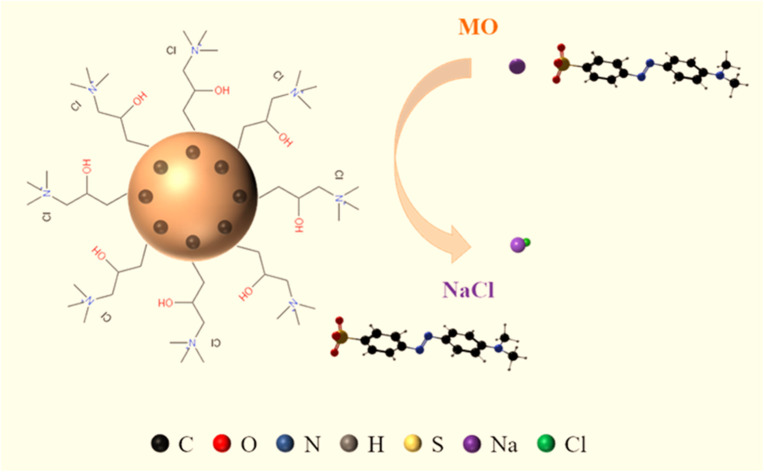

## 1 Introduction

Energy scarcity and environmental pollution represent two major challenges to human development. Industry's rapid growth has led to the consumption of most non-renewable resources. This consumption leads to the emission of various pollutants, seriously polluting the Earth's limited water resources. In the field of environmental protection, removing toxic and harmful chemicals from water has become a crucial task.^1–4^

Natural polymer materials such as chitosan, biochar, and alginates are considered excellent carriers for adsorbing pollutants in water treatment.^5–8^ Compared with traditional carriers, natural polymer materials have advantages such as low cost, low toxicity, excellent biocompatibility, and high availability.^9–11^ Chitosan is widely recognized as a highly efficient biopolymer, known for its exceptional adsorption capacity, stability in the presence of metal ions, and strong attraction to transition metals.^12–16^ Chitosan is derived from chitin through a deacetylation process. Chitin is a linear amino polysaccharide that occurs widely in the wings or shells of arthropods.^17^ The amine and hydroxyl functional groups in the chitosan molecular structure possess exceptional bioactivity, enabling it to exhibit remarkable biological functions and the ability for chemical modification reactions.^18–20^ Due to the protonation of its amino groups, chitosan tends to dissolve in acidic solutions, limiting its widespread application in water treatment. To overcome this limitation, various cross-linking agents were used to crosslink the amino and hydroxyl groups on the chitosan chain, thereby enhancing its chemical stability and versatility.^21,22^ However, due to cross-linking reactions, the amino and hydroxyl groups on chitosan are partially consumed, which reduces their adsorption capacity to various harmful substances.

The modification of chitosan can alter its chemical and physical properties, enhancing its applicability for water treatment.^23–26^ Quaternized chitosan is a novel functional material with potential applications among chitosan derivatives.^27^ Grafting quaternary ammonium groups onto chitosan molecules can increase the positive charge density on the chitosan surface. Quaternized chitosan retains the excellent adsorption capacity, biocompatibility, and biodegradability of chitosan, while exhibiting quaternary ammonium salts' antibacterial and moisturizing properties.^28,29^ This makes chitosan widely applicable in pharmaceuticals, antibacterial agents, moisturizers, catalysts, and bi-material carriers.^30^

Fe_3_O_4_ NPs are a common iron-based magnetic material. It is widely used in many fields due to its simple preparation, stable oxidative activity, and easy recovery.^31–33^ When combined with chitosan, they form composite microspheres that possess both the physicochemical properties of chitosan and the magnetic responsiveness of Fe_3_O_4_ NPs. Currently, magnetic chitosan microspheres have found extensive applications in the fields of food, medicine, and water treatment.^34–36^ These composite microspheres are used in protein adsorption and immobilization, enzyme purification, organic acid extraction, and removal of dye molecules and heavy metal ions.^37–39^ The application of an external magnetic field allows for the rapid separation of these composite microspheres from the solution, effectively preventing secondary contamination. After desorption, they can be reused, which is significant in terms of environmental protection and cost savings.^40^

In this study, the quaternized magnetic chitosan microspheres were prepared as adsorbents, and their adsorption performance was investigated. The obtained adsorbents were characterized using SEM, FTIR, XRD, and XPS. The effect of the synthetic condition on the adsorption performance of the adsorbent was discussed, and the adsorption kinetics and isotherm models of the microspheres for methyl orange were studied.

## 2 Experimental details

### 2.1 Materials

Chitosan (CS, with a deacetylation degree of 80.0–95.0%), glacial acetic acid, liquid paraffin, span-80, glutaraldehyde (GA, with a concentration of 25%), trisodium citrate, iron(iii) chloride hexahydrate (FeCl_3_·6H_2_O), and anhydrous sodium acetate were all supplied by China Pharmaceutical Group Chemical Reagent Co. Ltd. (Shanghai, China). Ethyl acetate (EA, with a concentration of 99.5%) was purchased from Shanghai Aladdin Bio-Chem Technology Co. Ltd. (Shanghai, China). Glycidyl trimethyl ammonium chloride (GTMAC,≥95%) was acquired from Shanghai McLean Biochemical Technology Co. Ltd. (Shanghai, China). Ethylene glycol, petroleum ether (60–90), and anhydrous ethanol were purchased from Xilong Scientific Co. Ltd. (Guangdong, China). Methyl orange (MO) was provided by Shanghai Runjie Chemical Reagent Co. Ltd. (Shanghai, China). All reagents used in the experiments were analytical grade and used as received.

### 2.2 Preparation of Fe_3_O_4_

Fe_3_O_4_ nanoparticles were prepared through the following procedure.^41^ Firstly, 0.46 g of trisodium citrate and 1.08 g of FeCl_3_·6H_2_O were added to 40 mL of ethylene glycol. The mixture was stirred continuously at 80 °C for 0.5 h to form a dispersion solution. Subsequently, 2.4 g of anhydrous sodium acetate was added to the above solution. The mixed solution was stirred for 0.5 h at 80 °C and then transferred to a sealed polytetrafluoroethylene reactor at 200 °C for 12 h. The reactor was cooled to room temperature. The black precipitate was collected using a magnetic block and repeatedly rinsed with deionized water and ethanol. The obtained black powder was dried at 60 °C in a vacuum for 12 h.

### 2.3 Preparation of quaternized magnetic chitosan

0.50 g of Chitosan was added to 20 mL of acetic acid solution (wt.2%) and stirred to form a uniform solution. Then, the obtained Fe_3_O_4_ was ultrasonically dispersed in the CS solution. 60 mL of liquid paraffin and an appropriate amount of Span 80 were added to a three-necked flask, stirring the mixture for 0.5 h. Subsequently, the Fe_3_O_4_/chitosan solution was introduced into the three-necked flask and heated to 45 °C with continuous stirring for 1.0 h. An appropriate amount of glutaraldehyde was slowly introduced into the mixed system, which was then heated to 60 °C and stirred for 3.0 h. The magnetic chitosan (MCS) was washed sequentially with anhydrous ethanol, petroleum ether, and deionized water several times, and then dried at 50 °C for 12 h.

**Scheme 1:**
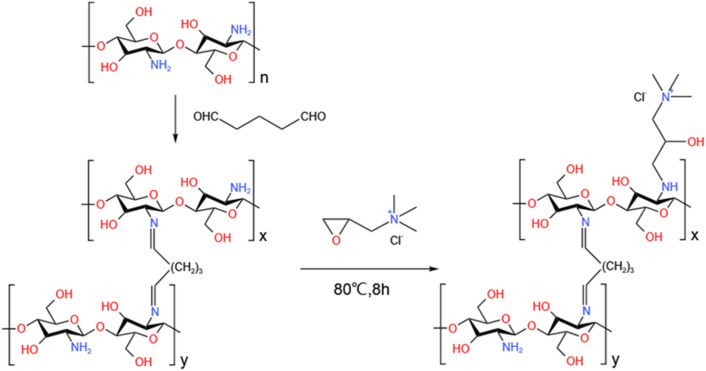
Synthesis route of QMCS.

A solution was prepared by mixing 1.97 g of GTMAC with 50 mL of deionized water in a flask. Subsequently, 0.46 g of MCS was introduced into the solution of quaternary ammonium salt, and the resulting mixture was heated to 80 °C for 8 hours under stirring. The product was rinsed multiple times with anhydrous ethanol and deionized water. Finally, the products were dried at 50 °C for 12 h to obtain quaternized magnetic chitosan (QMCS). The synthesis route of QMCS was illustrated in Scheme 1.

### 2.4 Characterization

The scanning electron microscopy (SEM) images were observed by Zeiss Merlin compact field emission with an accelerating voltage of 10 kV. Fourier transformed infrared (FT-IR) of CS, MCS, QMCS, and QMCS-MO was collected with a Nicolet IS 10 (ThermoFisher) with the wavenumber range of 4000 to 500 cm^−1^. X-ray diffraction (XRD) patterns were determined using an XRD-6000 X-ray diffractometer (Shimadzu, Japan) with a CuKα radiation source in the 2*θ* range of 10–90° at a scan speed of 5°/min. UV-vis absorption spectra were measured using an L8 spectrophotometer (Shanghai Yidian, China) with a wavelength range from 200 to 800 nm. X-ray photoelectron spectra (XPS) were recorded using a PHI5800 X-ray photoelectron spectroscopy analyzer (ULVCA-PHI, USA).

### 2.5 Adsorption experiments

The adsorption experiments of QMCS with MO were carried out in 100 mL round-bottom centrifuge tubes. 20 mg of QMCS was put into 80 mL of the methyl orange solution with 125 mg L^−1^. The centrifuge tubes were placed in an oscillator (150 rpm) at a preset temperature, and the supernatant of the reaction was periodically collected. The absorbance was measured using a UV-visible spectrophotometer at the wavelength of maximum absorption of MO. The effects of pH (4–10), contact time (1–360 min), and temperature (15, 25, 35 °C) on the adsorption efficiency of MCS and QMCS were systematically investigated. The pH of the methyl orange solution was adjusted using 0.1 M HCl and 0.1M NaOH solutions. These data were used to calculate the adsorbent's adsorption capacity and removal efficiency. Moreover, a series of experiments were conducted to investigate the effect of pH value and adsorbent dosage on adsorption performance, as well as adsorption kinetics and adsorption isotherms. The relevant formulas and parameters used in these experiments are provided in Table S1 of the ESI.†

### 2.6 Regeneration of the sorbent elution

To desorb the adsorbed MO, the adsorbent after adsorption was immersed in 0.1 M NaOH for 6 h. The adsorbent was collected with a magnet, rinsed with anhydrous ethanol and deionized water, then vacuum dried at 60 °C for 6 h. The regenerated adsorbents were cycled five times under the same adsorption and desorption conditions. The conditions for the adsorption experiment were as follows: the initial concentration of MO of 100 mg L^−1^, the initial pH of 5, the adsorption temperature of 298 K, and the adsorption time of 12 h.

## 3 Results and discussion

### 3.1 Characterization

#### 3.1.1 SEM

**Fig. 1:**
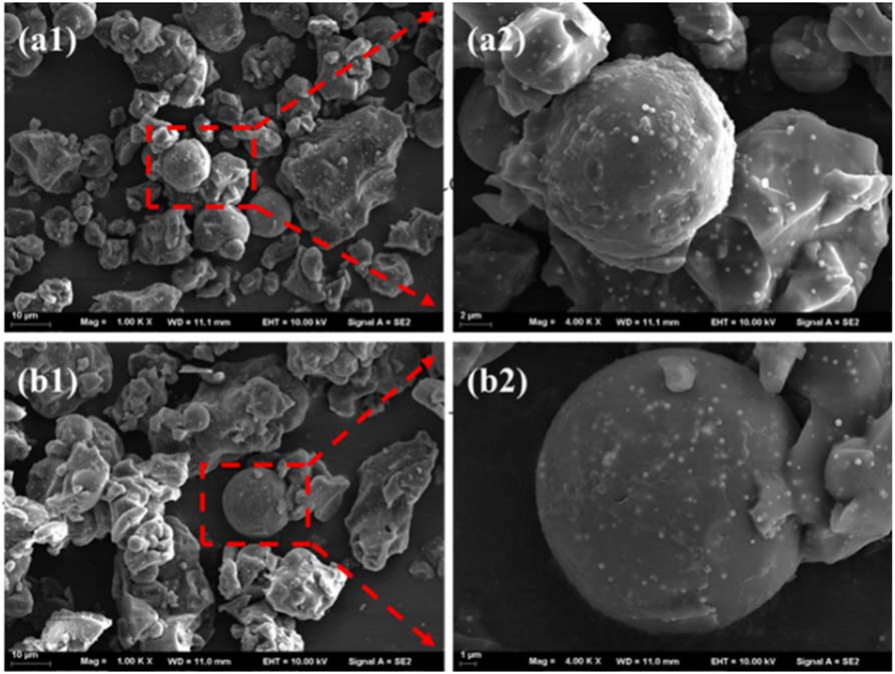
SEM images of MCS (a1, a2) and QMCS (b1, b2).

The SEM images of MCS and QMCS are shown in Fig. 1. It can be observed that the chitosan is a spherical particle, and its size is about 3–15 μm. The surface of spherical particles has an irregular shape, and Fe_3_O_4_ nanoparticles are uniformly anchored on the surface of MCS, as shown in Fig. 1(a2).^42^ In Fig. 1(b1-b2), it can be observed that the surface structure of the QMCS exhibited no significant changes compared to the MCS. The Fe_3_O_4_ nanoparticles are still anchored on both the surface and inside of the microspheres. The irregular surface of MCS and QMCS microspheres increases their specific surface area and provides more adsorption sites for the adsorption of pollutants.^43^

#### 3.1.2 FTIR analysis

**Fig. 2:**
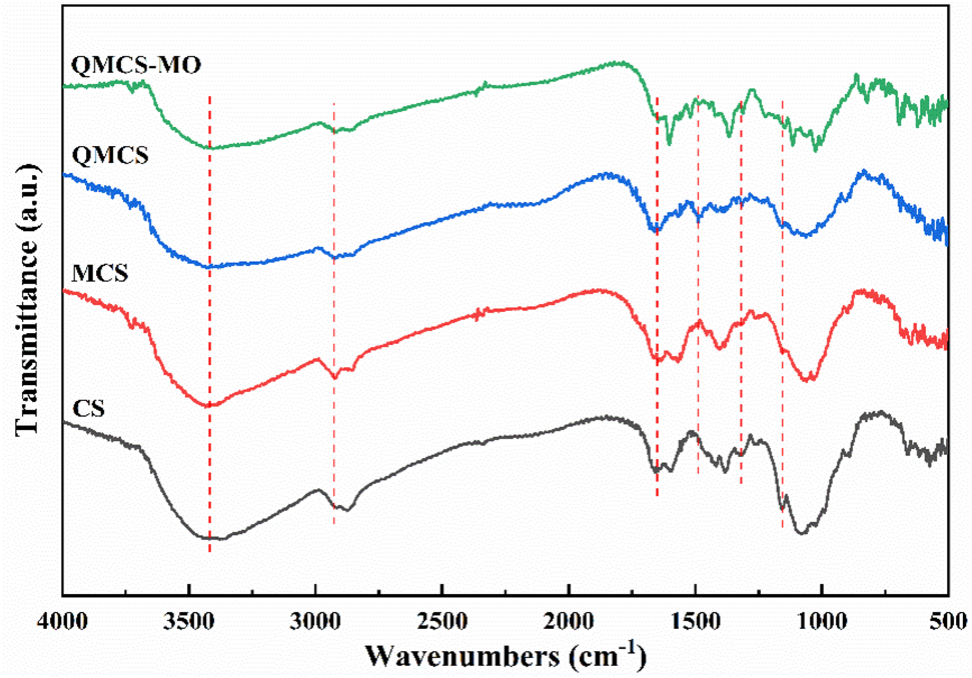
FT-IR spectra of CS, MCS, QMCS, and QMCS-MO.

The FT-IR spectra of CS, MCS, QMCS, and QMCS-MO (QMCS adsorbed with MO) are described in Fig. 2. The peak at 3433 cm^−1^ is the stretching vibration of O–H. The absorption peaks at 2920 cm^−1^ and 2876 cm^−1^ belong to the stretching vibration peaks of the –CH_2_ and –CH_3_ groups. The absorption peak at 1650 cm^−1^ is attributed to the stretching vibration peak of C=O, and the peak at 1150 cm^−1^ belongs to the stretching vibration peaks of C–O–C. The absorption peak at 1598 cm^−1^ belongs to the bending vibration of the N–H of the amide group, and the peak at 1318 cm^−1^ in the CS belongs to the stretching and bending vibration of C–N and N–H of the amide group. The absorption peak at 1560 cm^−1^ of MCS was attributed to the formation of an imine bond resulting from the nucleophilic addition reaction between glutaraldehyde and the amide groups.^29^ The intensity of absorption peaks at 1560 cm^−1^ and 1318 cm^−1^ exhibited a decrease, which can be attributed to the depletion of the amide groups during the crosslinking reaction.

For QMCS, the absorption peak at 1560 cm^−1^ can be observed to disappear, which is attributed to the quaternary ammonium salt grafted onto the chitosan molecule chain. A new absorption peak appears at 1488 cm^−1^, attributed to the C–H bending vibration in the quaternary ammonium salt.^44^ The peak of the amide group in QMCS at 1318 cm^−1^ showed a significant weakening. The changes indicated that GTMAC has successfully grafted onto MCS through the amide groups. For QMCS-MO, the peak at 1488 cm^−1^ exhibited a considerable decrease. The absorption peaks at 1605 cm^−1^ and 1025 cm^−1^ correspond to the stretching vibration of N=N and the stretching vibration of S=O, respectively.^45^ This indicated that the quaternary ammonium salt in QMCS participated in the adsorption process.

#### 3.1.3 XRD

**Fig. 3:**
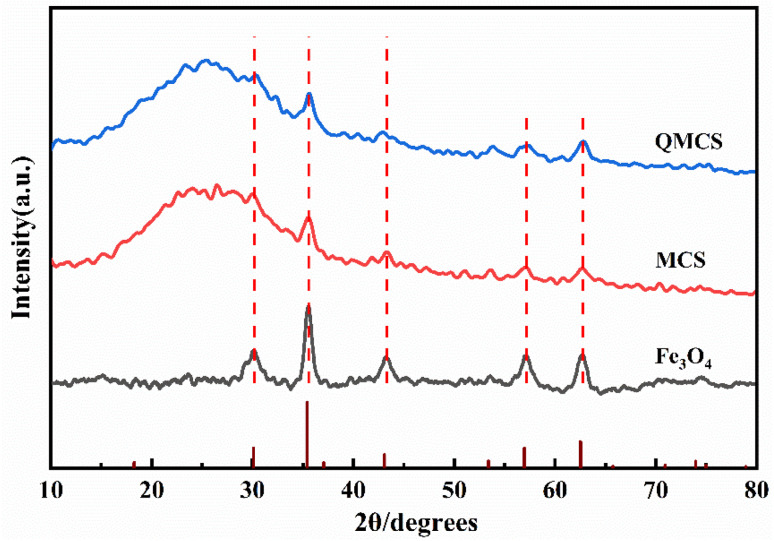
The XRD patterns of Fe_3_O_4_, MCS, and QMCS.

The XRD pattern of Fe_3_O_4_ was recorded in Fig. 3. The peaks at 18.42°, 30.14°, 35.58°, 37.14°, 43.26°, 53.64°, 57.10°, and 62.74° corresponded to the (111), (220), (311), (222), (400), (422), (511), and (440) crystal planes of Fe_3_O_4_ (JCPDS card no. 19-0629), respectively.^34^ XRD pattern of MCS confirmed the presence of Fe_3_O_4,_ which indicated that the Fe_3_O_4_ mixed well with chitosan during the preparation of MCS. The peaks of QMCS at 30.28°, 35.58°, 42.96°, 53.72°, 57.12°, and 62.6° suggested that quaternary ammonium modification of MCS does not affect the structure of Fe_3_O_4_.

#### 3.1.4 XPS

**Fig. 4:**
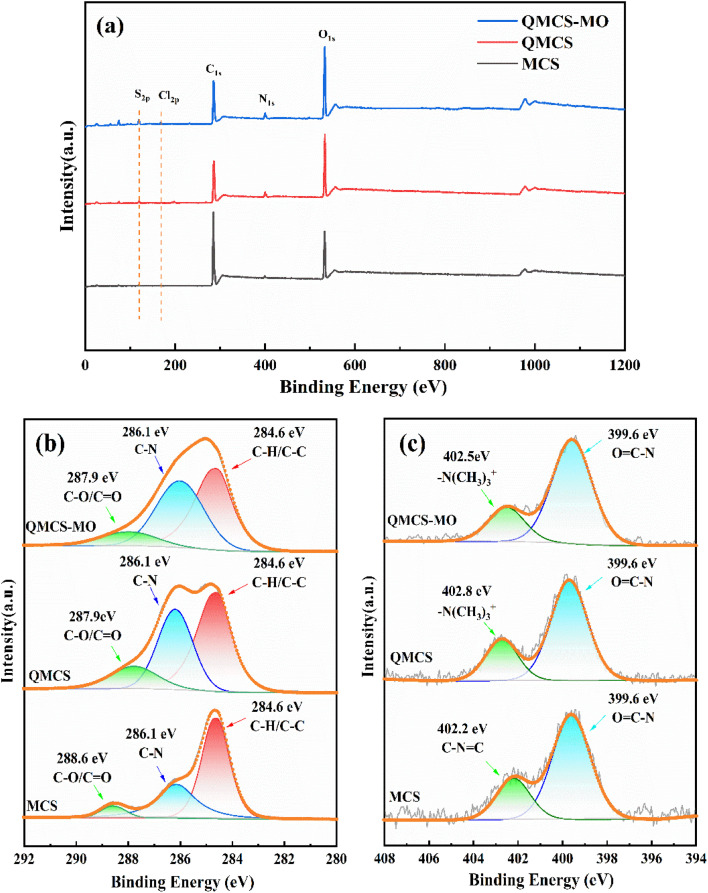
The full-scale XPS of MCS, QMCS, and QMCS-MO (a), high-resolution XPS spectra of C 1s (b) and N 1s (c) for MCS, QMCS, and QMCS-MO.

X-ray photoelectron spectroscopy (XPS) was used to examine the surface chemical state and elemental composition of the samples. Fig. 4(a) shows the elemental composition of MCS, QMCS, and QMCS-MO, including S, Cl, C, N, and O. Comparing the XPS full spectra of MCS and QMCS, the N peak in QMCS showed a significant increase compared to that in MCS. Additionally, a new Cl peak appeared at 198.1 eV, indicating the successful composite formation of GTMAC with MCS. Comparing the XPS full spectra of QMCS and QMCS-MO, it can be observed that the adsorption of MO led to a significant decrease in the Cl peak at 198.1 eV in QMCS, accompanied by the emergence of the S peak at 168.1 eV. This indicates that an exchange occurred between the Cl^−^ ions of the quaternary ammonium salt and the -SO_3_^−^ of MO during the adsorption process of MO by QMCS.

**Fig. 5:**
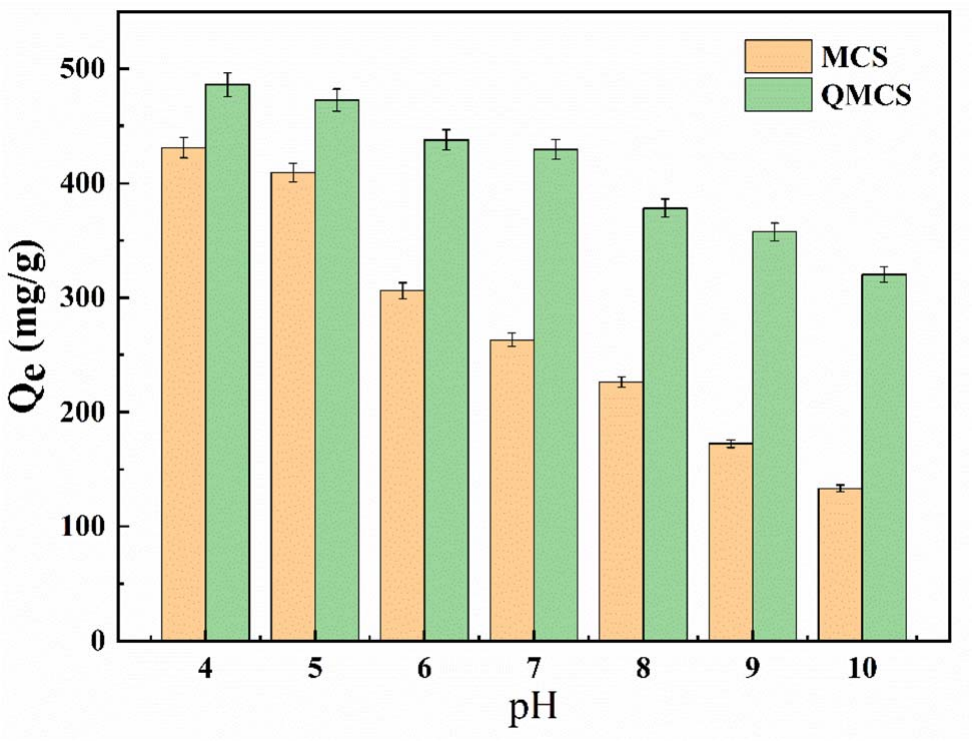
Effect of solution pH on adsorption capacity of MCS and QMCS.

Fig. 4(b) represents the C 1s spectra for MCS, QMCS, and QMCS-MO. The high-resolution spectra of C 1s in MCS show three peaks located at 284.6 eV, 286.1 eV, and 288.6 eV, representing the C–H and C–C, C–N, and C–O/C=O, respectively.^32^ In QMCS, a significant enhancement was observed in the C–N peak at 286.1 eV, and the peak of C–O/C=O shifted from 288.6 eV to 287.9 eV. For QMCS-MO, the peaks at 284.6 eV, 286.1 eV, and 287.9 eV remained unshifted, suggesting that the C–H, C–C, C–N, and C–O/C=O in QMCS did not undergo any reactions during the adsorption process of MO. Fig. 5(c) displays the N 1s spectra for MCS, QMCS, and QMCS-MO. The high-resolution spectra of the N 1s in MCS show two peaks at 399.6 eV and 402.2 eV, representing the O=C–N and C–N=C.^19^ A slight shift of the N 1s peak to 402.6 eV was observed in QMCS, which can be attributed to the introduction of –N(CH_3_)^3+^ during the grafting process. For QMCS-MO, a shift in the peak of the quaternary ammonium group was observed from 402.8 eV to 402.5 eV, indicating the involvement of the quaternary ammonium group in the adsorption reaction of MO by QMCS.

### 3.2 Adsorption performance

#### 3.2.1 Effect of pH

The pH value of the solution has a significant effect on the adsorption capacity of the adsorbent. The effect of pH value on the adsorption capacity of MCS and QMCS is illustrated in Fig. 5. When the pH value gradually increases from 4 to 10, the adsorption capacity gradually decreases. At an initial pH value of 4, the maximum adsorption capacities of MCS and QMCS were observed to be 431.49 mg g^−1^ and 486.13 mg g^−1^, respectively. The amino groups of chitosan undergo protonation, forming -NH_3_^+^ groups in acidic conditions. This enhances the electrostatic adsorption of sulfonic acid groups present in methyl orange with MCS. As a result, with an increase in pH value, the adsorption efficiency of MCS for methyl orange decreased.^29^ Therefore, as the pH decreases, the electrostatic repulsion between the positive charges on the QMCS surface and the cations of methyl orange weakens the adsorption effect. As the solution pH increases, the protonation of amino groups decreases gradually, and simultaneously, OH^−^ competes with MO for adsorption sites on MCS through ion exchange. Consequently, with the increase of the pH value, the adsorption performance of MCS for methyl orange gradually decreases.

Quaternization modification introduces positively charged -N(CH_3_)_3_^+^ groups on the surface of chitosan through the introduction of quaternary ammonium. This modification resulted in chitosan microspheres retaining more surface positive charges at higher pH values than their unmodified counterparts. Consequently, the quaternization modification enhanced the adsorption capacity of chitosan microspheres for anionic dyes at higher pH values.^46^ Furthermore, with the decrease in pH, the adsorption capacity of QMCS for MO also increased, indicating the presence of unmodified amide groups on QMCS. Under acidic conditions, these groups undergo protonation, enhancing the electrostatic adsorption capability of QMCS for MO. QMCS exhibits excellent adsorption performance under all test conditions, indicating its significant potential for application in capturing anionic dyes in aqueous solutions.

#### 3.2.2 Effect of adsorbent dosage

**Fig. 6:**
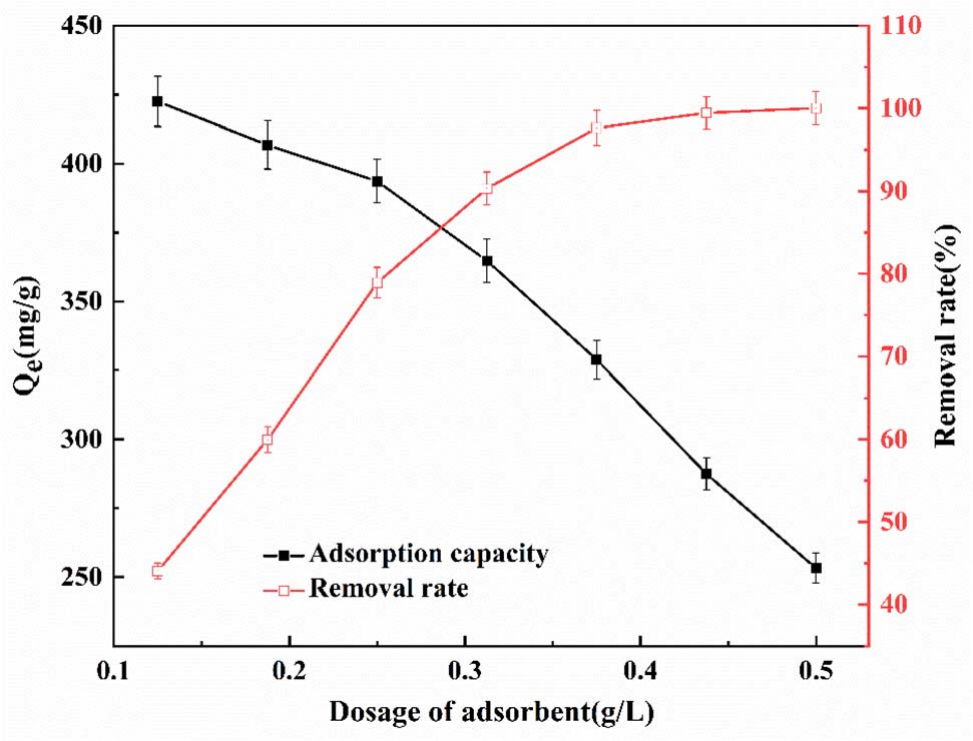
Effect of the dosage of QMCS on the adsorption of MO.

To understand the interaction between MO in the solution and the adsorption sites of QMCS, it is necessary to determine the optimal dosage of the adsorbent.^47^ The effect of the QMCS dosage on the MO's adsorption capacity and removal rate is illustrated in Fig. 6. It was evident that, with an increase in dosage, there was a substantial improvement in the removal efficiency of MO. With the increase of QMCS dosage from 0.125 g L^−1^ to 0.375 g L^−1^, the removal efficiency for MO was significantly enhanced. Subsequently, when the QMCS dosage increased from 0.375 to 0.5 g L^−1^, the removal efficiency reached a plateau. However, as the adsorbent dosage increases, the adsorption capacity of QMCS decreases. This is because as the dosage of adsorbent increases, the available adsorption sites also increase, leading to an improvement in removal efficiency. As the adsorbent absorbs all the methyl orange, the number of unoccupied active adsorption sites will increase. As a result, the adsorption capacity decreases gradually with an increase in the adsorbent dosage.^48^

### 3.3 Adsorption kinetic models

**Fig. 7:**
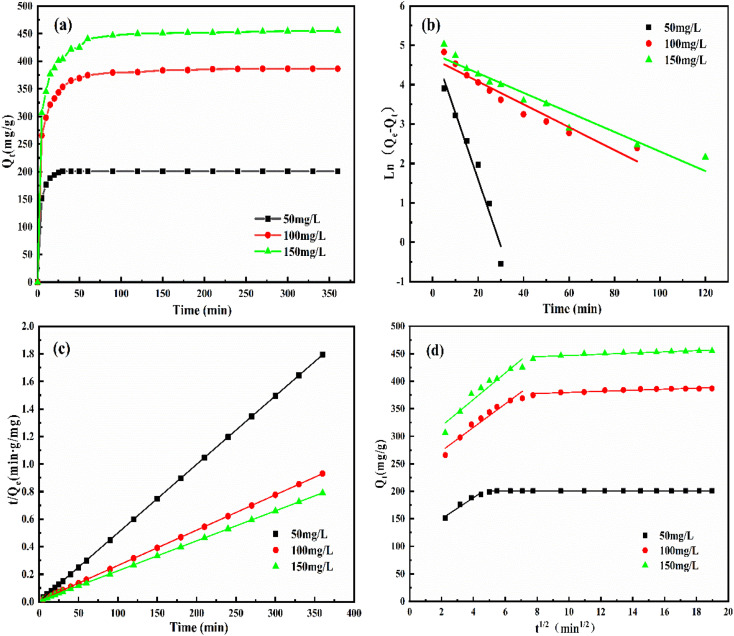
(a) Adsorption kinetics curve of MO by QMCS at different concentrations, (b) pseudo-first-order kinetic model, (c) pseudo-second-order kinetic model, and (d) particle diffusion model.

Fig. 7(a) illustrates the adsorption kinetics of QMCS for MO at different concentrations. With the increase of adsorption time, the adsorption capacity of QMCS continuously rises. During the initial 30 minutes, QMCS exhibited a relatively fast adsorption rate for MO. As the adsorption time increased, the adsorption rate gradually slowed down. As the adsorption time increased from 30 to 120 minutes, the adsorption rate of QMCS towards MO gradually decreased, while the adsorption capacity slowly increased. When the adsorption time exceeds 120 minutes, the QMCS exhibits minimal variation in the adsorption rate of MO, suggesting that the system has attained adsorption equilibrium and the adsorption capacity has stabilized. The results suggested that during the initial stages of adsorption, the surface of QMCS microspheres presented a substantial number of active adsorption sites that effectively bound with methyl orange. However, with the extension of the adsorption time, these adsorption sites gradually became saturated, resulting in a gradual decrease in the adsorption rate. After 120 minutes of adsorption, the equilibrium state was reached. The equilibrium adsorption capacity of QMCS in MO solutions with concentrations of 50, 100, and 150 mg L^−1^ was 200.63 mg g^−1^, 386.48 mg g^−1^, and 455.34 mg g^−1^, respectively.

The kinetic data were analyzed using a quasi-first-order kinetic model, a quasi-second-order kinetic model, and an intraparticle diffusion kinetic model to provide an interpretation. The fitting results are shown in Fig. 7 (b–d) and Tables S1 and S2.† The analysis results indicate that the quasi-second-order kinetic equation is more suitable for the adsorption process of methyl orange by QMCS. In addition, the adsorption capacity obtained from adsorption experiments is closely related to the equilibrium adsorption capacity calculated through fitting. This indicates that the adsorption of QMCS for methyl orange followed a chemical adsorption process, suggesting the existence of interactions such as ion exchange and chelation reactions between QMCS and methyl orange.^49^

Based on the analysis of Fig. 7(d) and Table S2,† it can be observed that the fitting line of the particle diffusion model for the adsorption kinetics data exhibits a two-stage division. This suggests that the adsorption process followed multiple stages and was not solely influenced by the adsorption rate. The fitting lines for both the first and second stages were linear and did not pass through the origin, suggesting that the adsorption of MO on QMCS, in addition to intra-particle diffusion, was also influenced by the boundary layer effect. This suggests that with the increase in the initial concentration of MO, the diffusion performance within the particles is enhanced. Furthermore, as the initial concentration of MO increased and the diffusion boundary layer concentration also increased, it indicated that the boundary layer effect strengthened gradually with the rising dye concentration.^50^ As most adsorption sites for MO on QMCS were occupied, the MO already adsorbed on QMCS hindered the diffusion of the remaining MO, making it difficult to further adsorb substances with higher concentrations.

### 3.4 Adsorption equilibrium isotherm

**Fig. 8:**
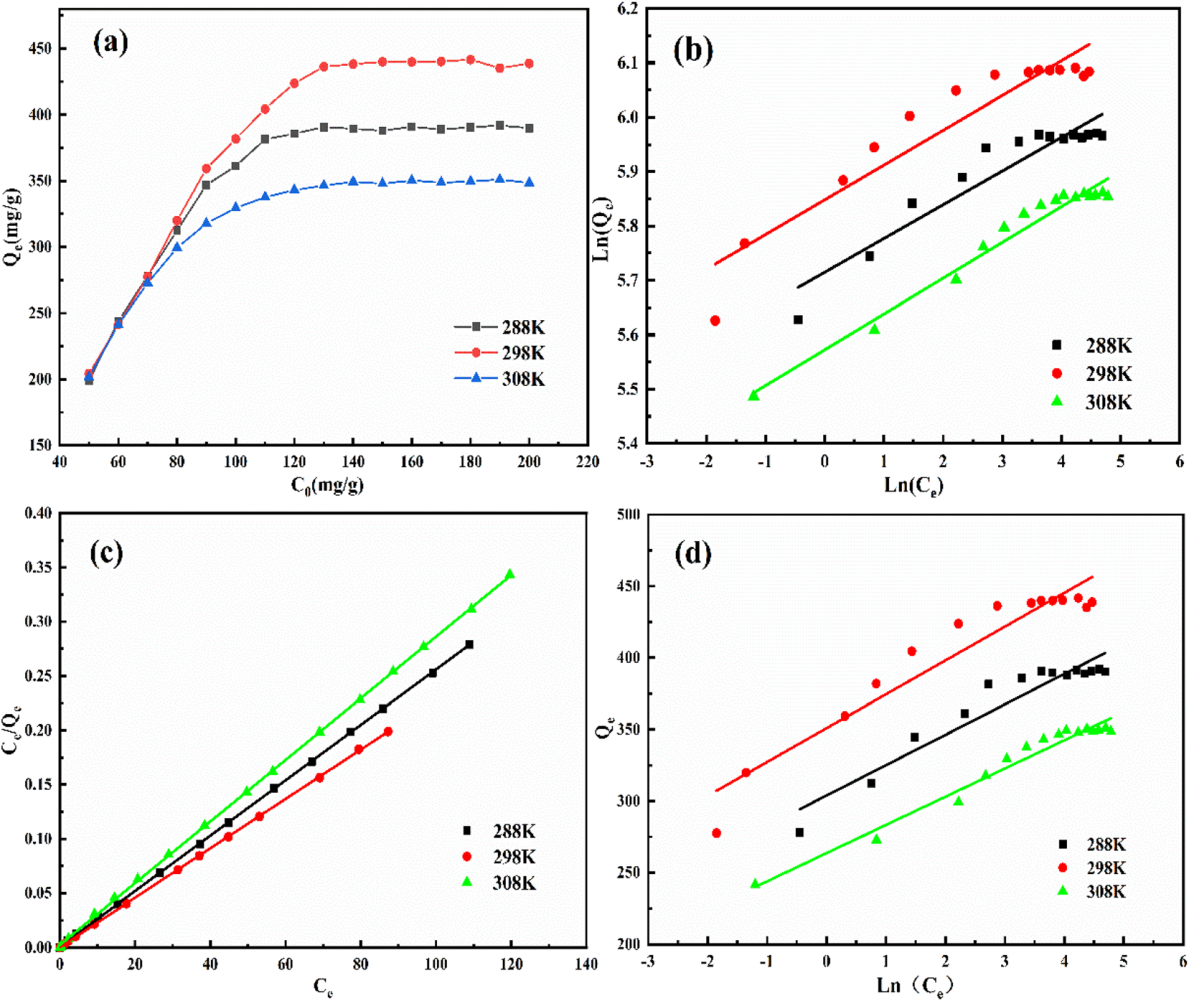
(a) Isothermal adsorption curve of MO by QMCS, (b) Freundlich isotherm model fitting curve, (c) Langmuir isotherm model fitting curve, and (d) Temkin isotherm model fitting curve.

Fig. 8(a) represents the isothermal adsorption curves of QMCS at 288 K, 298 K, and 308 K. As the concentration of MO increased from 50 mg L^−1^ to 120 mg L^−1^, the adsorption capacity of QMCS for MO gradually increased. When the MO concentration reached 130 mg L^−1^, the adsorption capacity of the adsorbent approached saturation. Additionally, as the concentration increases from 130 mg L^−1^ to 200 mg L^−1^, the adsorption capacity does not show significant changes. QMCS exhibited enhanced adsorption performance for MO as the temperature rose from 288 K to 298 K. This is due to the acceleration of molecular motion under higher temperatures, thereby enhancing the adsorption capacity of QMCS. However, with a further increase in temperature from 298 K to 308 K, a decline in the adsorption performance was observed. This suggests that as temperature increases, the desorption rate of MO from QMCS exceeds the adsorption rate, resulting in a decline in adsorption efficiency.

The isothermal adsorption data were fitted to the Freundlich isotherm model, the Langmuir isotherm model, and the Temkin isotherm model. The fitting curves and detailed data are presented in Fig. 8 (b–d) and Table S3.† The *R*^2^ value of the Langmuir isotherm model is higher than that of the Freundlich and Temkin isotherm models. This indicates that the adsorption of MO by QMCS can be better explained by the Langmuir isotherm model, suggesting that the adsorption process for MO by QMCS is primarily monolayer adsorption.^51^ Through calculations, the maximum adsorption capacities of QMCS for MO at 288 K, 298 K, and 308 K were 392.16 mg g^−1^, 440.53 mg g^−1^, and 352.11 mg g^−1^, respectively.

### 3.5 Mechanism of adsorption

From the FT-IR spectrum of QMCS adsorbing MO, the absorption peak of the quaternary ammonium groups at 1488 cm^−1^ exhibited a significant decrease. The absorption peaks corresponding to the N=N and S=O bonds within the MO molecule appeared at 1605 cm^−1^ and 1025 cm^−1^, respectively. It indicates that during the adsorption process, quaternary ammonium groups participate in the adsorption reaction. XPS full-spectrum analysis reveals that the Cl peak of QMCS at 198.1 eV shows a significant decrease after conducting MO adsorption experiments. Moreover, an S peak is observed at 186.1 eV, indicating an ion exchange reaction between Cl^−^ in the quaternary ammonium salt and -SO_3_^−^ in MO during the adsorption process. In the N 1s energy spectrum of QMCS and QMCS-MO, the binding energy peak of N(CH_3_)_3_^+^ in QMCS-MO is observed to shift from its original position of 402.8 eV to 402.5 eV. This indicates that the quaternary ammonium group has a significant effect on the adsorption of MO, suggesting its involvement in the adsorption reaction.

In the adsorption experiments with varying pH, the adsorption performance of QMCS on MO initially exhibited an increasing trend, followed by a decrease as the pH increased. This indicates the presence of unreacted amide groups on QMCS microspheres, which undergo ammonium protonation in acidic conditions, thereby enhancing the adsorption capacity of QMCS for MO. By analyzing the adsorption kinetic curves at different MO concentrations, it was observed that the adsorption of QMCS on MO conforms more closely to a pseudo-second-order kinetic model. This suggests that the interaction between QMCS and MO involves a chemical adsorption process.

**Fig. 9:**
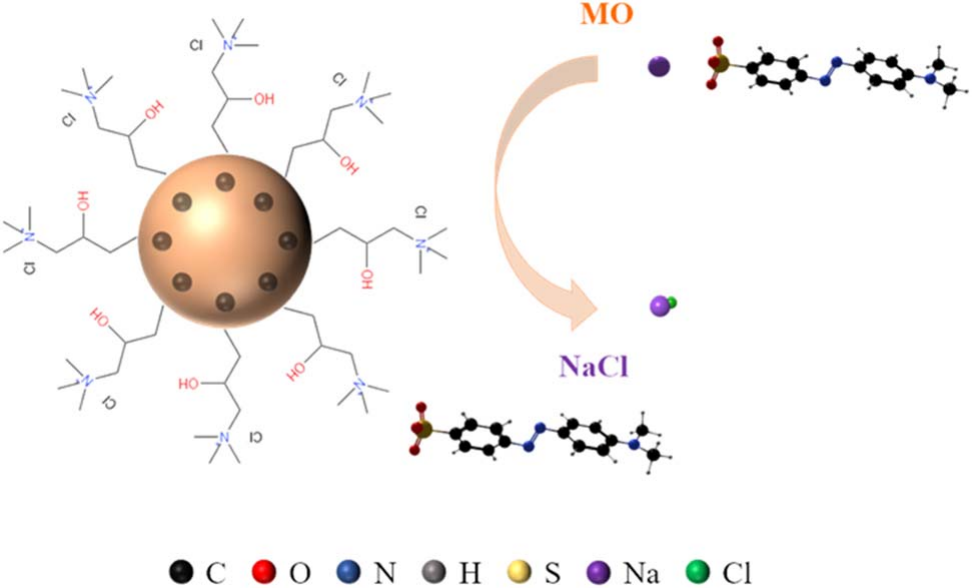
Adsorption mechanism.

Based on the preceding analysis, the adsorption mechanism of QMCS on MO is illustrated in Fig. 9. In aqueous solution, MO dissociates into Na^+^ ions and molecules with -SO_3_^−^. In the aqueous environment, quaternary ammonium groups (-N(CH_3_)_3_^+^) on QMCS and some protonated amide groups (-NH_3_^+^) play a crucial role in capturing MO through electrostatic interactions and ion exchange.^43,52^

### 3.6 Regeneration

**Fig. 10:**
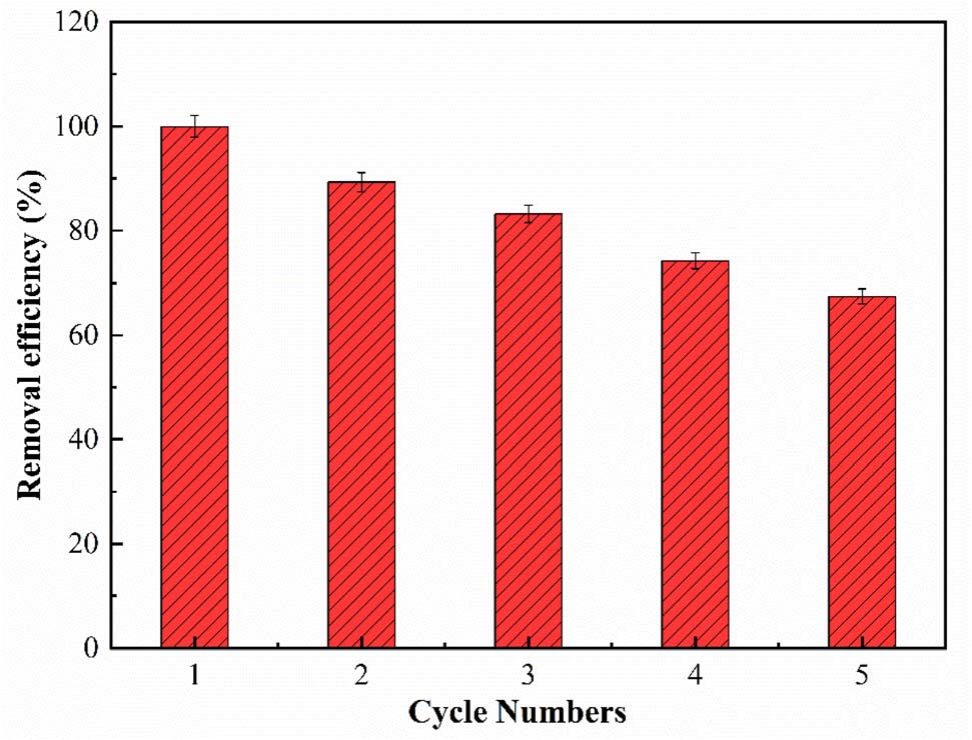
Removal efficiency of QMCS in 5 cycles.

The regenerative performance of the materials is an important factor in determining their actual applicability. The regeneration process of QMCS for MO was carried out using 0.1M NaOH as the eluent. Fig. 10 shows the removal efficiency of QMCS throughout 5 cycles. With the increase of the cycle number, the adsorption capacity of QMCS to MO gradually decreases. This was attributed to the utilization of NaOH solution, which displaced MO adsorbed on QMCS *via* an ion exchange mechanism involving OH^−^. However, MO remained on QMCS through van der Waals forces and hydrogen bonding.^29^ When adsorbed samples are regenerated using NaOH, a fraction of MO molecules may remain adsorbed due to stronger interactions, occupying active sites on the QMCS surface. With repeated cycling, the cumulative occupation of these adsorption sites results in a progressive reduction in removal efficiency. After 5 cycles, the removal efficiency of QMCS for MO was 67.46%, indicating that QMCS has good stability and regeneration performance.

The maximum adsorption capacity for MO is also compared with other reported adsorbents as summarized in Table 1. The QMCS exhibited excellent adsorption capacity (486.13 mg g^−1^) even with a low adsorbent loading (20 mg per 80 mL).

**Table 1:** The maximum adsorption capacities of different adsorbents for MO

Adsorbent	Dosage	pH	Maximum adsorption capacity (mg g^−1^)	References
Chitosan bead-like materials	70 mg per 100 mL	5–6	14.29	53
Chitosan-modified biochar	200 mg per 50 mL	3	38.75	54
Chitosan graft poly(acrylic acid)/graphite oxide/attapulgite	20 mg per 10 mL	3	186.5	55
Chemically crosslinked chitosan microspheres	30 mg per 60 mL	7	207	56
Guanidinium chitosan containing dicyclohexyl groups	50 mg	3	274	57
Chitosan/β-cyclodextrin	10 mg per 50 mL	5	392	58
Quaternized magnetic chitosan(QMCS)	20 mg per 80 mL	4	486.13	This work

## 4 Conclusion

Using emulsion cross-linking, quaternary ammonium salt modification resulted in quaternized magnetic chitosan microspheres. Compared to the unmodified adsorbent, the modified chitosan microspheres exhibited significantly enhanced adsorption capacity for MO. Adsorption kinetics and isotherm studies revealed that the adsorption of MO onto the modified chitosan followed a homogeneous monolayer chemical adsorption mechanism. In the adsorption experiment of MO on QMCS, electrostatic interaction and ion exchange play a significant role. These results demonstrate that the modified chitosan is an excellent adsorbent with outstanding adsorption performance for anionic dyes even under higher pH conditions and efficient reusability, providing a strong basis for practical applications.

## Author contributions

Kai Wang: data curation, formal analysis, methodology, writing – original draft. Zewen Song: data curation, formal analysis, writing – review & editing. Ziyi Xu: investigation, methodology. Yang Xi: formal analysis, investigation. Yuwei Cui: formal analysis, investigation. Haijun Zhou: data curation, funding acquisition, project administration, supervision, writing – review & editing.

## Conflicts of interest

The authors declare that they have no known competing financial interests or personal relationships that could have appeared to influence the work reported in this paper.

## Supplementary Material

RA-015-D5RA02862K-s001

## Data Availability

The authors affirm that the data supporting the findings of this study are included in the article. Additional data can be made available from the corresponding author upon reasonable request.
