# Supplementary material for: Synthesis of quaternized magnetic chitosan and adsorption performance for methyl orange from aqueous solution
Source: RSC Adv. 2025 Jun 23;15(26):21121–32. doi: 10.1039/d5ra02862k (PMC12183549; doi:10.1039/d5ra02862k)
Supplement: RA-015-D5RA02862K-s001 [file RA-015-D5RA02862K-s001.pdf]

Supplementary Information (SI) for RSC Advances.
This journal is © The Royal Society of Chemistry 2025

Supplemental Materials

For

Synthesis of Quaternized Magnetic Chitosan and Adsorption Performance for Methyl Orange from Aqueous Solution

Kai Wang, Zewen Song, Ziyi Xu, Yang Xi, Yuwei Cui, Haijun Zhou*

（School of Materials Science and Engineering, Jiangsu University of Science and Technology, Zhenjiang, 212100, China）

Correspondence author: Haijun Zhou, zhouhaijun@just.edu.cn.

Table S1. The formula used in the adsorption experiment.

Model	Equation	Model	Equation
Adsorption capacity	$Q_t = \frac{(C_0 - C_t)V}{m}$	Removal rate	$R = \frac{(C_0 - C_t)}{C_0} \times 100\%$
Adsorption capacity at equilibrium	$Q_e = \frac{(C_0 - C_e)V}{m}$	Freundlich model	$LnQ_e = LnK_F + \frac{1}{n}LnC_e$
Pseudo-first-order kinetic model	$Ln\ (Q_e - Q_t)\ = LnQ_e - K_1 t$	Langmuir model	$Q_e = \frac{Q_m C_e K_L}{1 + K_L C_e}$<br>$R_L$=1/ (1+$K_L C_0$)
Pseudo-second-order kinetic model	$\frac{t}{Q_t} = \frac{t}{Q_e} + \frac{1}{K_2 Q_e^2}$	Temkin model	$Q_e = \frac{RTLnA}{B} + \frac{RTLnC_e}{B}$
Particle diffusion model	$Q_t = K_W t^{1/2} + C$		

The meaning of the parameters in the formula:

C_0: the initial concentration of MO solution (mg/L).

C_t: the concentration of MO corresponding to time t (mg/L).

C_e: the concentration of MO after adsorption equilibrium (mg/L).

V: the volume of the Methyl Orange solution (L).

m: the adsorbent mass (mg).

Q_t: the adsorption capacity (mg/g).

Q_e: the equilibrium adsorption capacity (mg/g).

Q_m: the maximum adsorption capacity (mg/g).

K_1: the pseudo-first-order kinetic rate constant (min^{-1})

K_2: the pseudo-second-order kinetic rate constant (g/mg min).

K_W: the intramolecular diffusion coefficient ($mg/g{\cdot}min^{1/2}$).

K_L: Langmuir constant (L/mg).

R_L: Dimensionless constant separation factor.

K_F: Freundlich constant related to adsorption capacity.

n: Freundlich constant related to adsorption intensity.

t: the adsorption time (min).

A: the maximum binding energy during adsorption equilibrium.

B: the heat-related parameters associated with the adsorption.

Table S2 Kinetic parameters for the adsorption of MO onto QMCS.

Kinetic model	Parameters	50 mg/L	100 mg/L	150 mg/L
Pseudo-first-order kinetic model	K_1 (min^{-1})	0.169	0.029	0.025
	Q_e (mg/g)	144.555	105.670	119.969
	R^2	0.955	0.922	0.933
Pseudo-second-order kinetic model	K_2 (g/mg·min)	8.821×10^{-3}	0.882×10^{-3}	0.667×10^{-3}
	Q_e (mg/g)	201.207	390.625	458.716
	R^2	0.999	0.999	0.999
Particle diffusion model	K_W (mg/g·$min^{1/2}$)	19.190	21.352	24.052
	C (mg/g)	111.557	230.681	270.544
	R^2	0.942	0.933	0.905

Table S3 Isotherm parameters for the adsorption of MO onto QMCS.

Isotherm model	Parameters	288 K	298 K	308 K
Freundlich	n	16.095	15.637	15.190
	K_F	176.761	346.610	263.049
	R^2	0.883	0.882	0.965
Langmuir	Q_m (mg/g)	392.157	440.529	352.113
	K_L	2.729	6.842	1.136
	R^2	0.999	0.999	0.998
Temkin	A	1.682×10^6	2.864×10^6	6.415×10^5
	B	112.966	101.458	129.898
	R^2	0.898	0.910	0.965
